# cath-resolve-hits: a new tool that resolves domain matches suspiciously quickly

**DOI:** 10.1093/bioinformatics/bty863

**Published:** 2018-10-08

**Authors:** T E Lewis, I Sillitoe, J G Lees

**Affiliations:** 1 Department of Structural and Molecular Biology, UCL, Darwin Building, London, UK; 2 Department of Biological and Medical Sciences, Faculty of Health and Life Sciences, Oxford Brookes University, Oxford, Oxfordshire, UK

## Abstract

**Motivation:**

Many bioinformatics areas require us to assign domain matches onto stretches of a query protein. Starting with a set of candidate matches, we want to identify the optimal subset that has limited/no overlap between matches. This may be further complicated by discontinuous domains in the input data. Existing tools are increasingly facing very large data-sets for which they require prohibitive amounts of CPU-time and memory.

**Results:**

We present cath-resolve-hits (CRH), a new tool that uses a dynamic-programming algorithm implemented in open-source C++ to handle large datasets quickly (up to ∼1 million hits/second) and in reasonable amounts of memory. It accepts multiple input formats and provides its output in plain text, JSON or graphical HTML. We describe a benchmark against an existing algorithm, which shows CRH delivers very similar or slightly improved results and very much improved CPU/memory performance on large datasets.

**Availability and implementation:**

CRH is available at https://github.com/UCLOrengoGroup/cath-tools; documentation is available at http://cath-tools.readthedocs.io.

**Supplementary information:**

Supplementary data are available at *Bioinformatics* online.

## 1 Introduction

Accurately annotating protein domains is essential for a number of tasks such as genome annotation. Various resources exist for assigning domains to proteins, each with its own distinct philosophy and approach (e.g. Dawson et al., 2016; Finn *et al.*, 2017; Lam *et al.*, 2016). Predicting domains for a query protein typically involves scanning the amino acid sequence against domain libraries and then resolving the candidate matches to obtain a final set of non-overlapping domain assignments. The scans typically assign a score (e.g. bit-score or *e*-value) to the candidate matches and this can be used to prioritise strong matches in cases of domain overlaps.

The simple greedy approach (select the best hit, followed by the next non-conflicting best hit, etc.) has been shown to be outperformed by a method that seeks a global optimum, DomainFinder3 (DF3) (Yeats *et al.*, 2010). DF3 is also able to deal with discontinuous domains, which arise from domains’ insertions into other domains (meaning these domains do not have a single continuous region on the protein sequence, but have multiple starts and stops). However, DF3 is based on a graph-based, maximal-weighted-clique algorithm and it becomes increasingly slow and memory intensive for larger proteins. Similar problems, such as weighted interval scheduling, can be tackled with fast, optimal dynamic-programming algorithms. However, naïvely translating such algorithms to domain resolution would not account for discontinuous domains and so would disregard solutions in which one domain is inserted within the gap of another, discontinuous domain.

## 2 Materials and methods

In this work, we present cath-resolve-hits (CRH), a new tool that uses a dynamic-programming algorithm so that CPU/memory usage scales better with increasing problem size. CRH is implemented in C++ for speed and memory efficiency.

We have chosen to frame the problem such that the algorithm itself is a deterministic search for an unambiguously optimal solution and all choices and trade-offs are pushed into the generation of the input data. This separation encourages the sort of transparency, reproducibility and debuggability that has previously benefited approaches to other bioinformatics problems such as sequence alignment.

The algorithm maximizes the sum of the selected matches’ scores. Users may provide any positive scores with their input data, though CRH also provides default translations from HMMER (hmmer.org) bit scores or *e*-values to scores suitable for CRH. This provides tremendous flexibility without sacrificing ease-of-use. CRH also provides options that re-weight the scores to adjust the strength of preference for high scores or for long/short domains.

CRH allows for limited overlaps between matches by trimming the ends of domains’ segments (according to a user-configurable policy) before resolving them. CRH can handle data for multiple query proteins in a single input file. It does not require that the data be pre-grouped by query (but if notified of such pre-grouping, can exploit it to reduce run-time and memory usage). See Supplementary Material for details.

To assess CRH performance, we built a benchmark set by mapping known CATH v4.2 domains from PDB structures to UniProt sequences, using the SIFTS resource (Velankar *et al.*, 2013). To remove redundancy, we clustered the SIFTS-mapped protein sequences with CD-HIT at 70% sequence identity, choosing the longest protein sequence from each cluster. The final benchmark dataset consisted of 4738 protein sequences (see Supplementary Material for details).

For these UniProt sequences, we assessed each method’s ability to reconstruct the original CATH domain assignments using domain predictions from a library of HMMs derived from CATH v4.2 definitions (Lewis *et al.*, 2018).

Real-world domain assignment tasks typically involve low sequence identity between the known domain used to build the HMM model and the domain being predicted. To simulate this, we removed any HMM models built from seed domains with more than some specified percentage identity to the known CATH domains in the query sequence. We applied this at three levels of sequence-identity cut-off: 100%, 60% and 30%.

## 3 Results

**Fig. 1.**
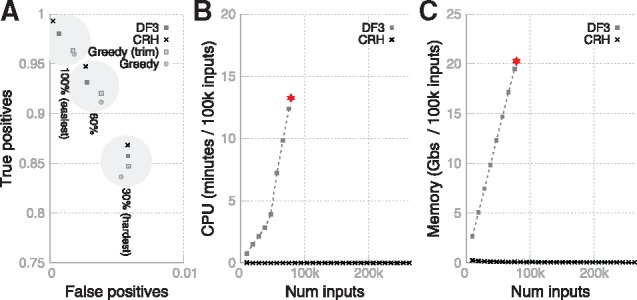
(**A**) performance of CRH, DF3 and Naïve Greedy at 100%, 60% and 30% sequence identity homology removal (see Methods). The axes show the proportion of domains assigned to: the correct domain superfamily (*y*-axis); an incorrect domain superfamily (*x*-axis). CRH assignments for all the Benchmark HMM assignments with 475, 161 hits took 3.3 s (Intel i7-7500U up to 3.5 GHz) and peak memory usage of 143 MB. A perfect result would appear at the top-left corner. B/C) Rate of use of CPU time in minutes (**B**)/memory in GBs (**C**) per 100 000 inputs to resolve a randomly chosen subset of hits to a large protein (human titin), averaged over 100 runs. The stars indicate the points beyond which DF3 failed to run, even with ample memory available

We found that CRH’s performance is very similar to or slightly better than DF3’s (Fig. 1A). Both methods exhibited overall improvement over naïve-greedy approaches (both with and without domain overlap trimming) (Fig. 1A).

A few sequences from this dataset were used in CRH’s development so we cannot exclude the possibility of overfitting, however contact was minimal and we think this is unlikely.

The main difference we found between CRH and DF3 was that CRH shows greatly improved memory efficiency and speed. We demonstrated this by measuring the time/memory that each program required to resolve random subsets of 263 312 Gene3D-v16 HMM predictions to the 34 350-residue TITIN_HUMAN sequence (Q8WZ42) on the same CentOS 6 machine (Fig. 1B and C). Each measurement was averaged over 100 runs. CRH appears to exhibit a constant rate of CPU/memory usage per input (hence linear growth overall), whereas DF3 appears to exhibit a linear rate of usage (hence quadratic overall). Further, DF3 crashed when run on any datasets of 84 636 models or more, even with ample memory provided. This shows CRH’s better suitability for tackling the enormous growth in biological data [illustrated by the tens of billions of sequences now available from the IMG/M resource (Markowitz *et al.*, 2012)].

CRH also provides greater flexibility in both input and output formats. Though DF3 and CRH both accept simple generic formats, CRH can also process both the raw and domain table outputs from hmmsearch and hmmscan (hmmer.org). Furthermore, there are several available output formats from CRH, including basic text, graphical HTML and JSON. The graphical HTML output (Supplementary Fig. S1) is useful for laying the domain resolution process bare, revealing why specified domains are included/excluded in the final resolved domain architecture.

CRH is available for Linux and Mac as part of a suite of tools at https://github.com/UCLOrengoGroup/cath-tools. The project is written in C++14. The code compiles without warning or error under strict settings of both GCC and Clang. Travis-CI is used for builds and for continuous-integration execution of >¼-million test assertions in >1000 test cases, with and without Clang’s AddressSanitizer.

## Funding

JGL was funded by BBSRC (Ref: BB/L002817/1).

*Conflict of Interest*: none declared.

## Supplementary Material

bty863_Supplementary_Material
