# Supplementary material for: cath-resolve-hits: a new tool that resolves domain matches suspiciously quickly
Source: Bioinformatics. 2018 Oct 8;35(10):1766–7. doi: 10.1093/bioinformatics/bty863 (PMC6513158; doi:10.1093/bioinformatics/bty863)
Supplement: bty863_Supplementary_Material [file bty863_supplementary_material.docx]

Supplementary Material

*
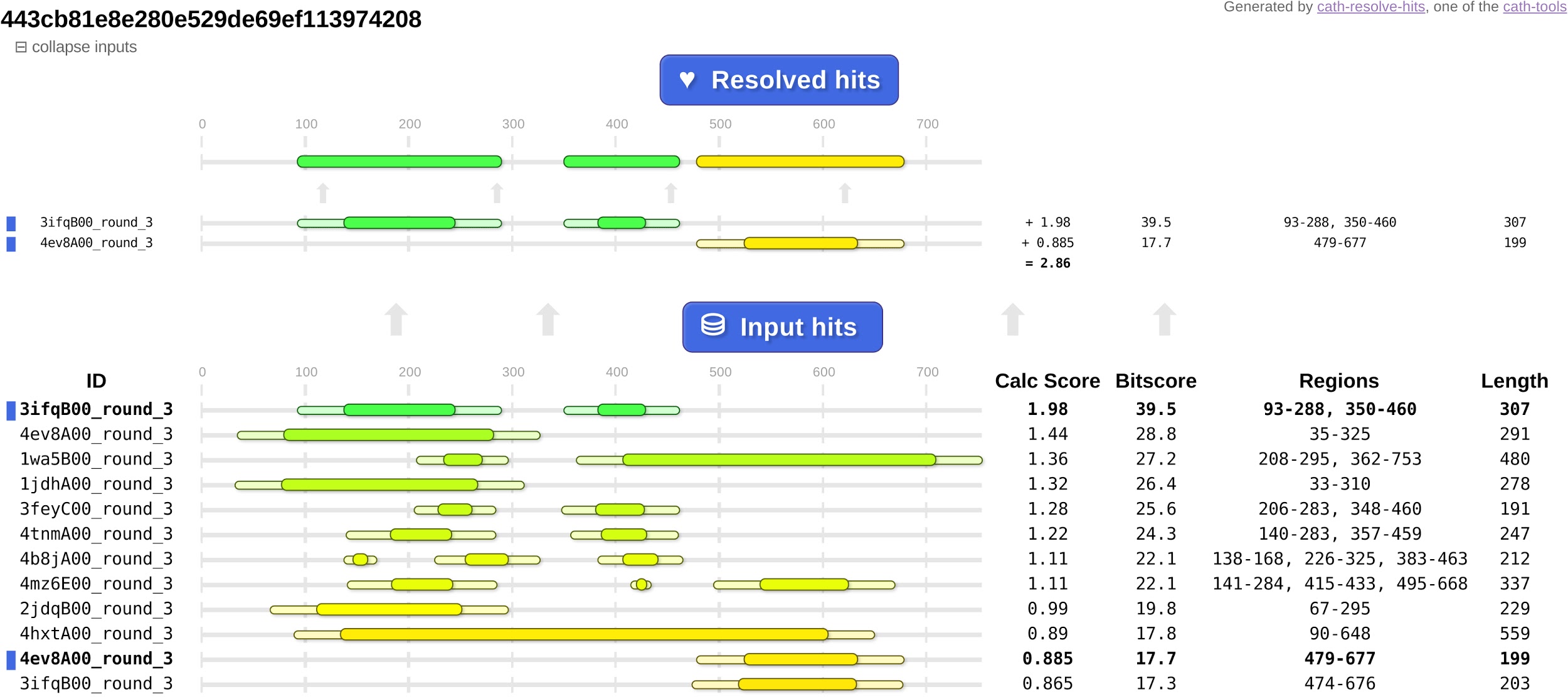
*

*Supplementary Figure 1. Example HTML output from cath-resolve-hits.*

## Dynamic Programming Approach

The following text refers to boundaries between residues rather than residues themselves because this makes things simpler to reason about (both in prose and in code). In these terms, a continuous domain can be thought of as running from the boundary at the start of one residue to the boundary at the end of another.

To begin with, consider a simplified version of the problem in which all domains are required to be continuous.

A naïve, brute-force search for the optimum for a data-set of n entries would involve checking 2^n^ combinations, meaning that even a moderate, thousand-hit example would require an absurd >10^300^ checks. Fortunately, we can do better. This simplified problem is equivalent to the established "Weighted Interval Scheduling" problem and can be tackled with the same dynamic-programming approach.

Dynamic-programming techniques involve reusing solutions to sub-problems to build towards a solution to the full problem. In our case, we can calculate the optimal solution for the region up to some boundary i much more easily if we already know the optimal solutions for all the regions up to the boundaries 1, 2, 3, ..., i-1. We can take the optimal solution for i-1 and check whether we can beat its score with any of the architectures that include a domain that stops at i. And we can calculate all of these optimal architectures for domains that stop at i by combining each such domain with the optimal architecture up to the domain's start boundary, which must be one of the known optimal solutions.

This strategy for calculating each next step allows us to iteratively build from nothing to the optimal solution for the whole sequence. If we invest O(n . log n) time on pre-sorting the n domains in the input, then we can calculate the optimal architecture in O( n ) time using this approach.

*
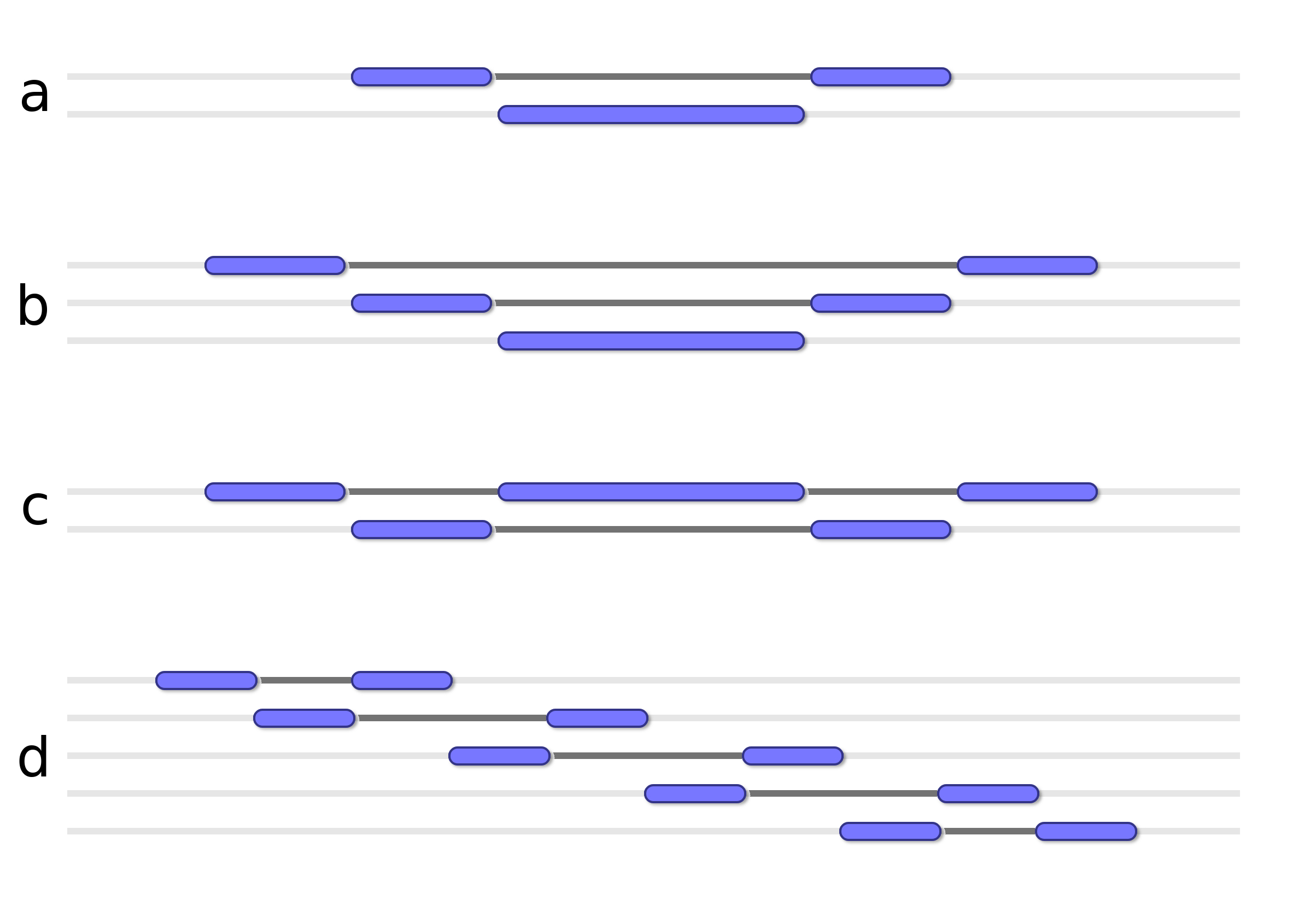
*

*Supplementary Figure 2. Illustrations of the levels of complexity the algorithm must handle: (a) one domain inside the interior gap of a discontinuous domain (b) a discontinuous domain inside another (c) a discontinuous domain spanning multiple interior regions of another (d) a pattern of interspersing that can trigger back-chaining.*

## Handling Discontinuous Domains

We can now remove this model’s simplifications in stages. First, let's introduce discontinuous domains that can include continuous domains within their interior gap regions (Supplementary Figure 2a). This makes things harder: when evaluating some boundary i and calculating the optimal architecture associated with one of the domains that stops there, we must now recognise that this domain may be discontinuous. In that case, we must also factor in the optimal way to fit domains inside the discontinuous domain's interior region(s). To calculate that, we can reuse the same dynamic-programming technique within that interior region.

The next stage introduces discontinuous domains that sit inside one interior region of another discontinuous domain (Supplementary Figure 2b). This can be handled by a process of recursion: on encountering a discontinuous domain, recurse one layer deeper into a new dynamic-programming scan to find the optimal solution within its interior region(s).

Next we can introduce discontinuous domains that span multiple interior regions of another discontinuous domain (Supplementary Figure 2c). This can be handled by making each recursive call specify a mask that marks out those regions already occupied by domains in higher levels of recursion and hence defines the available regions for which this layer must determine the optimal, fitting architecture. This mask can be incorporated into each dynamic-programming scan by simply rejecting any candidate domains that overlap with it. When the current layer of recursion encounters a discontinuous domain, it can pass to the new level of recursion a copy of the mask it was using, modified to incorporate the new discontinuous domain's regions.

## Preventing Back-Chaining

The final (and trickiest) stage is to introduce non-nested, interspersed discontinuous domains. In principle, these can be tackled with a recurse-with-mask approach, similar to that described above, in which each level of recursion may now be required to consider regions before those considered by the level before. Unfortunately this leaves the algorithm vulnerable to terrible running-times when the data includes any groups of hits like: 1 2 1 3 2 4 3 5 4... (Supplementary Figure 2d) because these will induce a sort of back-chaining in the algorithm. For example, calculating the result up to domain 5 will involve evaluating domain 4, which will involve recursing into domain 4 and performing the same amount of work as was involved when initially evaluating domain 4. This reinstates O( 2^n^ ) running times for any sub-groups of input domains like these.

We can prevent this back-chaining by exploiting an observation: the computation for evaluating domain 4 whilst inside domain 5 is identical to the earlier one for initially evaluating domain 4 *up to the boundary at the start of domain 5*. Hence, we can avoid duplicating this computation by detecting this situation during the initial processing of domain 4 whilst passing the boundary at the start of domain 5, and caching the best result seen so far. We can index the cache of this best result under the unmasked regions for which it provides the known optimal solution. Then when we later re-encounter 4 whilst processing within 5, we must still recurse inside 4 but can now re-use all the work done up to the start of 5. This approach guarantees that each layer of recursion tackles a smaller region than the layer before and hence prevents back-chaining.

The algorithm’s recursive structure means we could still construct data sets on which it would be slow. This could be tackled with additional caching of results for discontinuous domains' interiors. However we haven’t added this as we have found the existing algorithm to be consistently very fast across the large data sets on which we've tried it.

## Benchmarking Details

We built a benchmarking set by mapping known CATH v4.2 domains from PDB structures to UniProt sequences, using the SIFTS resource (Velankar et al, 2013). CATH domains were only included if all or all-but-one of their residues could be mapped to the corresponding UniProt sequence. To remove redundancy, we clustered the SIFTS-mapped protein sequences with CD-HIT at 70% sequence identity, choosing the longest protein sequence from each cluster. Before running the benchmark we removed mapped domains without a homologous superfamily assignment.

Running BLAST showed some CATH domain sequences from different superfamilies had >70% sequence identity and we filtered the benchmarking to remove the effects of these cross-hits along with some manual filtering to remove additional problems in the benchmark dataset mainly arising from CATH annotation conflicts that were evident after mapping to UniProt with SIFTS. These examples lead to mis-predictions by both DF3 and CRH even since the error was not with the methods themselves, but with the benchmark dataset, these accessions were removed (P09732, Q9QUH6, Q9Y618, Q9Y0H4, Q13625). A number of HMMs in the benchmarks showed strong cross-hits between superfamilies which affected the domain annotations for UniProt accessions (O28603, Q8IAR7, P34164, H7C745) and these were also removed from the benchmark set.

We then assessed how effectively CRH and DF3 could recreate the sequences’ original CATH domain architectures from the results of scans of the sequences against HMM models taken from Gene3D-v16. We used default parameters for DF3 and CRH. The domains were provided to both methods to only include those with e-values < 0.001 (from a search space defined form the initial scans (-Z parameter) of 10000000).

To better represent real world prediction tasks, we removed any HMM model built from a seed domain that had more than some specified percentage identity to the known CATH domains in the query sequence. We applied this at three levels of sequence-identity cut-off: 100%, 60%, and 30%, which we calculated using BLAST-P. Note that for a given benchmark protein and a given sequence-identity cut-off, if there were no HMMs remaining from the same superfamily as a benchmark domain we were attempting to predict we removed that benchmark domain from that sequence-identity cut-off benchmark set. When assessing true positives and false positives, we compared the family assignments of the prediction domain and benchmark domain if they overlapped by at least 50%.
